# Development of Lateral Flow Assay Based on Size-Controlled Gold Nanoparticles for Detection of Hepatitis B Surface Antigen

**DOI:** 10.3390/s16122154

**Published:** 2016-12-16

**Authors:** Dong Seok Kim, Yong Tae Kim, Seok Bok Hong, Jinwoon Kim, Nam Su Heo, Moon-Keun Lee, Seok Jae Lee, Byeong Il Kim, In Soo Kim, Yun Suk Huh, Bong Gill Choi

**Affiliations:** 1 Department of Chemical Engineering, Kangwon National University, Samcheok 25913, Korea; nyongtory91@gmail.com (D.S.K.); skychojja@gmail.com (S.B.H.); 2 Nanobio Application Team, National NanoFab Center (NNFC), Daejeon 34141, Korea; ytkim@nnfc.re.kr (Y.T.K.); mklee@nnfc.re.kr (M.-K.L.); sjlee@nnfc.re.kr (S.J.L.); 3 Department of Biological Engineering, Inha University, 100 Inha-ro, Nam-gu, Incheon 22212, Korea; anlfkrn2000@gmail.com (J.K.); bioheo6@gmail.com (N.S.H.); 4 Research and Development Center, Tomorrow & Solution (T&S), E19-604, 291 Daehak-ro, Yuseong-gu, Daejeon 34141, Korea; kbiset@gmail.com; 5 Research Center 2, Eudipia Inc., Cheongju 361-951, Korea; i.s.kim@eudipia.com

**Keywords:** gold, nanoparticle, hepatitis B surface antigen, lateral flow assay, conjugation

## Abstract

In this study, we developed lateral flow assay (LFA) biosensors for the detection of hepatitis B surface antigens using well-controlled gold nanoparticles (AuNPs). To enhance colorimetric signals, a seeded growth method was used for the preparation of size-controlled AuNPs with a narrow size distribution. Different sizes of AuNPs in the range of 342–137.8 nm were conjugated with antibodies and then optimized for the efficient detection of LFA biosensors. The conjugation stability was investigated by UV-vis spectroscopy of AuNP dispersion at various pH values and concentrations of antibody. Based on optimized conjugation conditions, the use of 42.7 ± 0.8 nm AuNPs exhibited superior performance for the detection of LFAs relative to other sizes of AuNPs.

## 1. Introduction

Hepatitis B virus (HBV) is a viral infection that can cause lifelong infection, hepatitis, liver cirrhosis, and liver cancer, resulting in about one million deaths each year. HBV, which can survive outside the human body for at least one week, is most commonly transmitted through contact with the blood or other body fluids of an infected person [1,2,3]. Basic markers for diagnosis of HBV infection include the presence of hepatitis B surface antigens (HBsAgs) and hepatitis B envelope antigens in acute or chronically infected hepatocytes [4,5]. Several physiological and biochemical methods have been developed to monitor HBV infection [6,7,8,9]. In addition, Abe et al. reported quantitative analysis of HBV using DNA Polymerase chain reaction (PCR) assay [10]. Although these methods provide accurate and sensitive detection of HBV, they require high-end instruments, a considerable amount of time, and skilled analysts. Accordingly, there is demand for the development of fast, simple, and sensitive diagnostic systems for point-of care HBV infection testing.

In clinical diagnosis, the importance of point-of-care (POC) testing techniques has led to the need for rapid, inexpensive, and highly efficient methods for the detection of disease biomarkers [11,12,13,14]. The lateral flow assay (LFA) method is a simple and powerful tool that can detect a variety of analytes from blood proteins to mycotoxins and from viral pathogens to bacterial toxins [15,16,17,18,19,20,21,22,23,24]. Most widely used LFA-based biosensors depend on changes in colorimetric signals that originate from the aggregation of colloidal gold nanoparticles (AuNPs) [15]. LFA biosensors are generally composed of a sample pad, conjugation pad, reaction membrane, and waste reservoir. The sensitivity of LFA biosensors is significantly influenced by the amounts of accumulated AuNPs captured on antibody-immobilized sites through sandwich-type immunoreactions. Several previous studies have reported that the diameter of the AuNPs influences the sensitivity of the AuNP-based immunochromatographic assay [15,16]. AuNPs sized 20–40 nm have been widely used in a variety of lateral flow assays. To enhance the sensitivity of LFA biosensors, the size of the AuNPs should be optimized with a narrow size distribution.

Herein, we synthesized AuNPs ranging in size from 34 nm to 137.8 nm with a narrow size distribution through a seeded growth method. As-prepared AuNPs were intensively investigated using a transmission electron microscope and dynamic light scattering analysis. Conjugation of antibodies and AuNPs was optimized by UV-vis spectroscopy of AuNP dispersions at various pH values and concentrations of antibodies. AuNP-based LFA biosensors with different-sized AuNPs were then fabricated for the detection of HBsAg. Among the different sizes of AuNPs, LFA biosensors using 42.7 nm AuNPs were found to be the most sensitive for the detection of HBsAg.

## 2. Materials and Methods

### 2.1. Materials

Gold(III) chloride trihydrate (HAuCl_4_·3H_2_O, 99%), trisodium citrate dihydrate, potassium phosphate monobasic (KH_2_PO_4_) and bovine serum albumin (BSA) were purchased from Sigma-Aldrich. Sucrose, potassium carbonate (K_2_CO_3_), Tween 20, disodium hydrogen phosphate (Na_2_HPO_3_·12H_2_O), and polyvinyl alcohol 1500 (PVA 1500) were purchased from Junsei Chemical Co., Ltd. (Tokyo, Japan). Affinity purified antibody against HBsAg, goat anti-mouse IgG, and recombinant HBsAg were purchased from Bore Da Biotech Co. Ltd. (Seongnam, Korea). Absorbent pad, backing card, nitrocellulose membrane (NC), sample pad, and conjugation pad were obtained from Bore Da Biotech Co. Ltd. (Seongnam, Korea).

### 2.2. Preparation of AuNPs

Different-sized AuNPs were synthesized by a seeded growth method [25]. First, Au seeds were prepared as follows. A sodium citrate solution (2.2 mM and 150 mL) was injected into three-necked round-bottomed flasks and then heated for 15 min, during which time the evaporation of the solution was blocked by a condenser. Then, 1 mL of HAuCl_4_ (25 mM) was added and reacted for 10 min. The color change was observed from yellow to dark pink. The as-prepared Au seed dispersion was kept at 90 °C. To control the size of AuNPs, 1 mL of sodium citrate (60 mM) and 1 mL of HAuCl_4_ (25 mM) were sequentially injected. After reaction for 30 min, the resultant product was AuNPs. This process was repeated up to 14 times to increase the diameter of AuNPs. After each cycle, samples were collected and characterized by transmission electron microscopy (TEM), UV-vis spectroscopy, and a dynamic light scattering (DLS).

### 2.3. Preparation of Antibody-Conjugated AuNPs

Prior to conjugating AuNPs with antibody, the pH of the AuNP dispersion was adjusted by adding 0.25 M K_2_CO_3_ solution. Antibodies were then added into the AuNP dispersion (5 mL), after which the mixture was incubated at room temperature for 30 min using a rotator. Following incubation, the mixture was centrifuged at 10,000 rpm for 15 min. The supernatant was then discarded, after which 1 mL of 1% BSA was added and the sample re-suspended. The mixture was subsequently incubated at room temperature for 30 min using a rotator. The centrifugation and suspension process were repeated twice, with 400 μL of BSA (1%) added in the final step. In order to optimize the conjugation process, the pH (6.17–10.0) and the concentration of antibody (1–20 μg/mL) were screened by measuring the minimum decrease of absorbance at OD_525_ after adding 1 wt.% NaCl solution.

### 2.4. Fabrication of Immunochromatographic Test Strip

The LFA strip was composed of a sample pad, conjugation pad, NC, and absorbent pad. Prior to assembly of the components, the conjgation pad was saturated with 0.05% PVA and 0.05% Tween 20 and dried at room temperature for 24 h. The conjugation pad was wetted by antibody-conjugated AuNP dispersion and dried at room temperature overnight in a desiccator, after which capture antibody (1 mg/mL) and goat anti-mouse IgG (1 mg/mL) were immobilized on the test line and control line of NC, respectively, using a dispersal system. The as-prepared sample pad, conjugation pad, NC, and absorbent pad were assembled on a backing card. A LFA strip was obtained by cutting the prepared card into 5 mm strips. To investigate the effects of AuNP size, AuNPs obtained after 1, 3, 7, 10, and 14 growing steps were used for fabrication of the LFA strips.

### 2.5. Measurement of Colorimetric Signal Intensity

The colorimetric signal from AuNPs on the test line was measured using an Immuno-strip Analyzer (T&S Co., Ltd., Daejeon, Korea) to quantify the amount of target analytes. Briefly, 100 μL of HBV antigen (conc. 500 ng/mL, 100 ng/mL, and 10 ng/mL) was loaded onto the sample pad, while antibody-conjugated AuNPs obtained after different growth steps of 1, 3, 7, 10, and 14 were sprayed onto the conjugation pad. The HBV antigen coupled with antibody-conjugated AuNPs on the conjugation pad, after which the AuNP linked HBV antigen was captured by antibody which was immobilized on the test line. The colorimetric signal was obtained using a Basler Ace camera (acA2500-14uc, Blaser, Ahrensburg, Germany) with the Aptina MT9P031 CMOS sensor (Blaser, Ahrensburg, Germany). A 60 W LED lamp was utilized with an analog controller (LS501A, Image Focus Company, Gwangmyeong, Korea) in the darkroom to create the constant lighting condition, while the strip images capturing. Captured images were processed via the Visual Inspector 2D software (Micro-Version, Cheonan, Korea), which is a home-made analysis program based on Visual C++.

## 3. Results and Discussion

**Figure 1:**
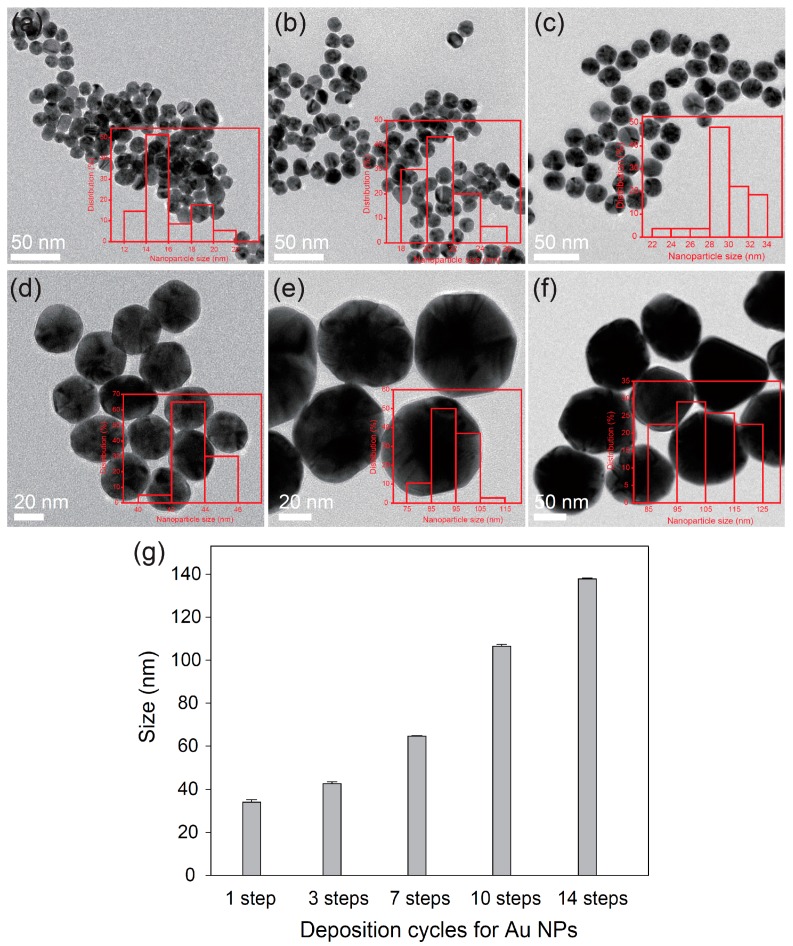
TEM images of (**a**) AuNP seeds and different-sized AuNPs according to growth steps; (**b**) 1 step; (**c**) 3 steps; (**d**) 7 steps; (**e**) 10 steps; and (**f**) 14 steps. Insets are size distribution diagrams for different-sized AuNPs. (**g**) AuNP sizes obtained from DLS.

**Figure 2:**
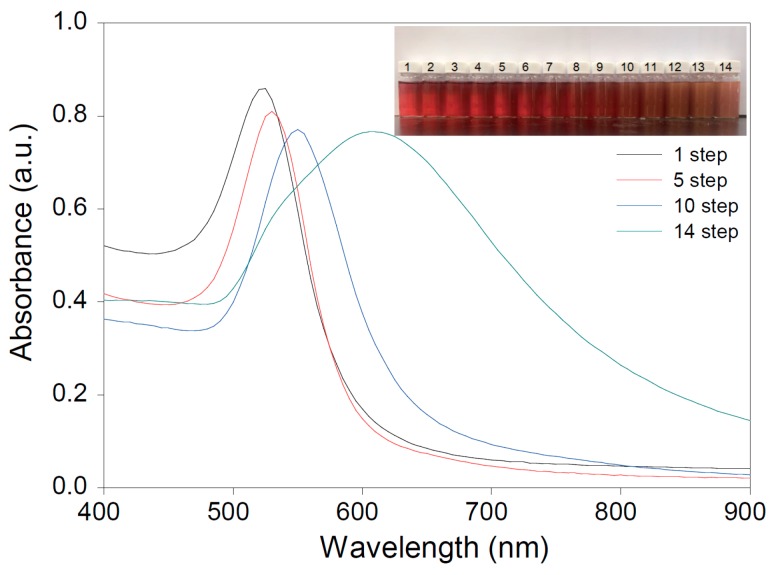
UV-vis spectra of dispersions of different-sized AuNPs. Inset is photograph of AuNP dispersions obtained from different seeded growth steps of 1 to 14.

Figure 1 shows typical morphologies of AuNPs obtained from different growth steps. As the growth step increased, the diameter of the AuNPs gradually increased, with values of 15.9 ± 2.5 nm (seeds), 21.7 ± 1.7 nm (1 step), 30.1 ± 2.5 nm (3 steps), 43.7 ± 1.2 nm (7 steps), 91.2 ± 8.1 nm (10 steps), and 105.9 ± 11.2 nm (14 steps), respectively (Figure 1a–f). In order to obtain the average size of AuNPs, DLS was measured (Figure 1g). The sizes were determined to be 34.0 ± 1.2 nm (1 step), 42.7 ± 0.8 nm (3 steps), 64.7 ± 0.2 nm (7 steps), 106.5 ± 0.1 nm (10 steps), and 137.8 ± 0.45 nm (14 steps), respectively. When observing high-resolution TEM images, a thin and conformal coating of the citrate capping agent was clearly observed on the surface AuNPs. The optical properties of the AuNP dispersion with different growth steps were investigated by UV-vis spectroscopy (Figure 2). The initial peak at 520 nm for AuNPs (1 step) showed red shifts and broadened as the size of the AuNPs increased from 34 to 137.8 nm. These results indicate that AuNPs had a quasi-sphere shape and uniform dispersion. The color of the AuNP dispersion changed from red to brownish-red as the size of the AuNPs increased (inset of Figure 2). Based on TEM and UV-vis spectroscopy, as-synthesized AuNPs exhibited a high quality with well-controlled NP sizes.

**Figure 3:**
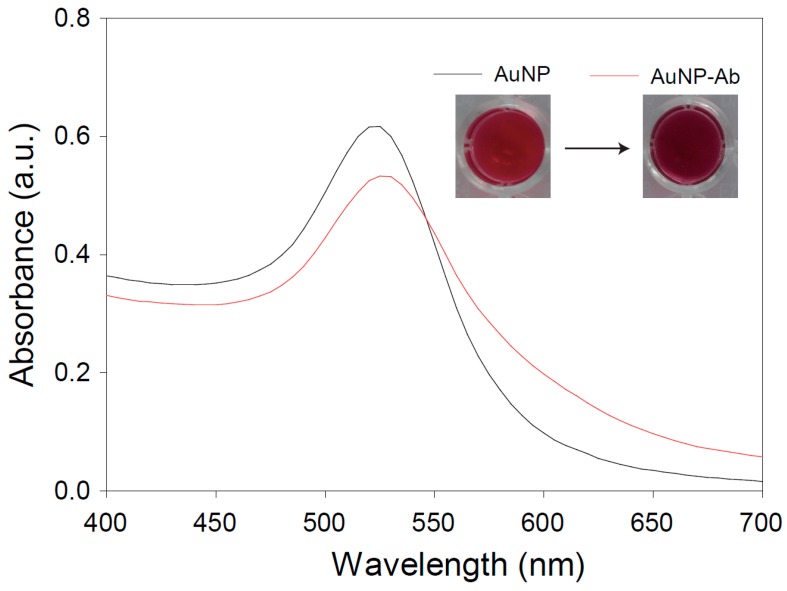
UV-vis spectra of AuNP and AuNP-Ab dispersions.

**Figure 4:**
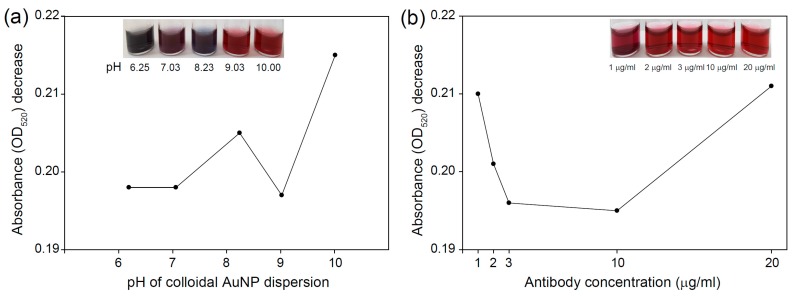

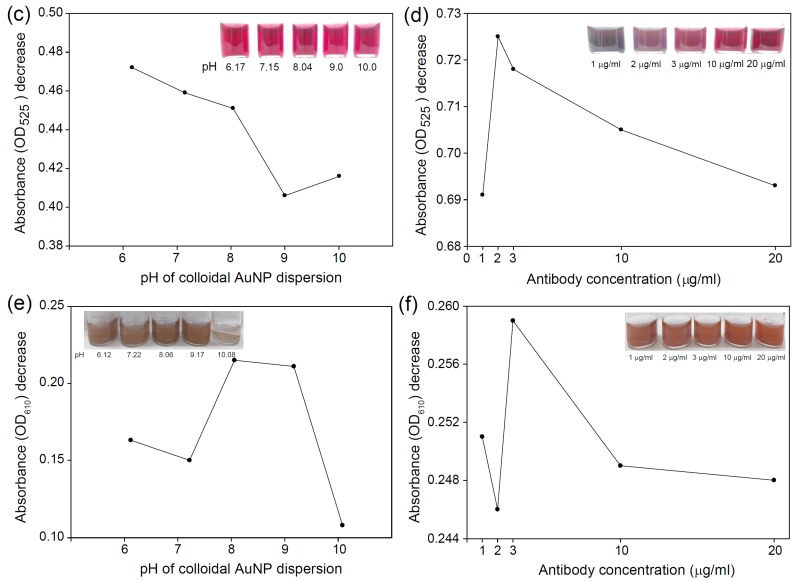
Changes in absorbance (OD_520, 525, and 610_) of the AuNP dispersion after adding NaCl at various pH values and at various concentrations of antibody after adding NaCl for different-sized AuNPs: (**a**,**b**) for 34.0 ± 1.2 nm AuNP; (**c**,**d**) for 42.7 ± 0.8 nm AuNP; and (**e**,**f**) for 137.8 ± 0.45 nm AuNP. Insets are photographs of the AuNP dispersions.

AuNPs conjugated with antibodies (AuNP-Ab) were characterized by UV-vis spectroscopy to investigate the conjugation stability and original color retention capability. The inset of Figure 3 shows photo images before and after Ab conjugation. Under optimized conditions, AuNP-Ab showed the same color AuNP dispersion. After conjugation with Ab, AuNP-Ab exhibited a gradual red shift and a decreased absorbance peak intensity (Figure 3). Exposure to high salt concentrations led to changes in the surface plasmon resonance of the colloidal AuNPs. AuNP flocculation could have occurred because of the high concentrations of electrolytes owing to the reduction in electrostatic repulsive charges between AuNPs. When the AuNPs aggregated, the color of the AuNP dispersion changed to grey or black. The flocculation assay was performed for the stable conjugation of AuNPs and Abs (Figure 4). Different-sized AuNPs with 34.0 ± 1.2 nm, 42.7 ± 0.8 nm, and 137.8 ± 0.45 nm were used. The optimized conjugation conditions were selected based on the minimum decrease of absorbance at OD_520, 525, and 610_ according to different pH values (around 6–10) and Ab concentrations (1–20 μg/mL) and the absence of apparent color changes from red to grayish-blue. Based on UV-vis spectroscopy and color changes, the optimum pH and concentration for AuNP-Ab (34.0 ± 1.2 and 42.7 ± 0.8) were 9 and 10 μg/mL, respectively (Figure 4a–d). When using larger-sized AuNPs (137.8 ± 0.45 nm), the optimum pH and concentration were changed to 7.22 and 20 μg/mL, respectively (Figure 4e,f).

**Figure 5:**
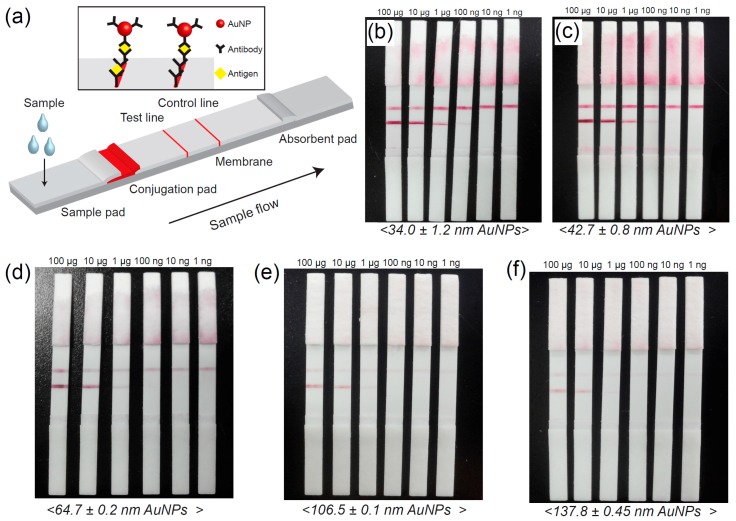
(**a**) Schematic illustration of LFA. Photographs of LFA test results based on different-sized AuNPs of (**b**) 1 step (34.0 ± 1.2 nm); (**c**) 3 steps (42.7 ± 0.8 nm); (**d**) 7 steps (64.7 ± 0.2 nm); (**e**) 10 steps (106.5 ± 0.1 nm); and (**f**) 14 steps (137.8 ± 0.45 nm).

**Figure 6:**
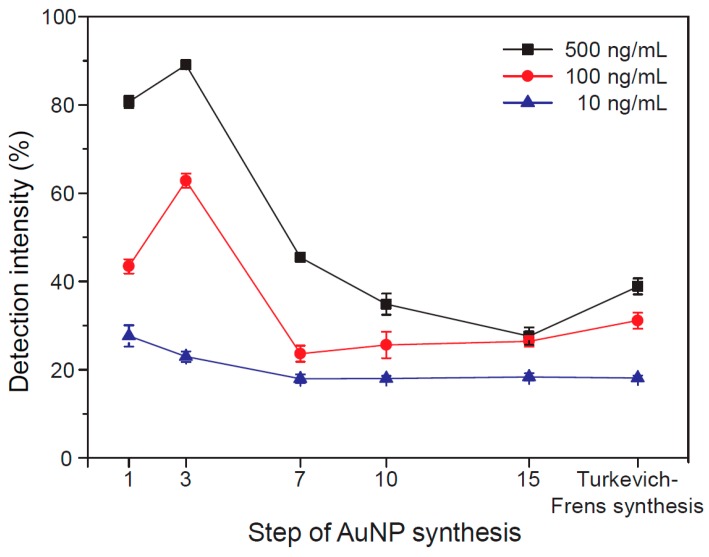
Color intensity at the test line of LFAs based on different-sized AuNPs and Turkevich-synthesized AuNPs.

We established LFAs with different sizes of AuNPs (1, 3, 7, 10, and 14 growth steps) based on optimized conjugation conditions to investigate the influence of nanoparticle size (Figure 5). Because the LFA test depends on many biological and non-biological components, we fabricated LFAs under the same experimental conditions while changing the size of the AuNPs. LFA based on AuNPs obtained from the Turkevich-Frens method was also tested as a control sample. LFAs were analyzed at antigen (Ag) concentrations of 1 ng, 10 ng, 100 ng, 1 μg, 10 μg, and 100 μg. Obviously, we observed the effect of AuNP size on the LFA performance. Specifically, 1 and 3 AuNP growth steps produced clearer results than LFAs based on 7, 10, and 14 growth steps. In addition, LFAs based on 1 and 3 growth steps could detect Ag concentrations as low as 1 ng. To investigate the influence of AuNP size on LFA performance, the color intensity at the test line of LFAs was measured using the Immuno-strip Analyzer (Figure 6). All intensities were detected after 10 min (Ag loading) and the recorded data were converted into percentages using the following equation:

(1) $$\mathrm{Detection}\;\mathrm{intensity}\;\left(\%\right)=\;\frac{V_{m}-{\;V}_{n}}{V_{c}{\;-\;V}_{n}}\;\times \;100$$

where *V*_c_ is the intensity of the control line of the negative control, *V*_n_ is the intensity of the bare nitrocellulose membrane, and *V*_m_ is the intensity of the test line from each sample. As shown in Figure 6, the detection intensity increased with the HBV concentration, and the LFA based on three growth steps (42.7 ± 0.8 nm) exhibited the best performance among LFAs. These signals of 42.7 ± 0.8 nm-based LFA were superior when compared to the LFA based on AuNPs obtained from the Turkevich-Frens method. These results indicate that the size of AuNPs plays an important role in the detection sensitivity [26,27]. The signal visibility of LFA could be improved by increasing the diameter of AuNPs from one to three steps because it resulted in increased observable differences in their size. However, the signal intensity decreased as the AuNP size increased from 3 to 15 steps, which may be attributed to the reduced conjugation efficiency of AuNPs. Larger-sized AuNPs (>42.7 ± 0.8 nm) needed a higher concentration of antibody (20 μg/mL). However, the use of a high concentration of antibody made the red color of AuNPs lighter (Figure 4), which resulted in the reduced signal intensity of LFAs (Figure 5).

**Figure 7:**
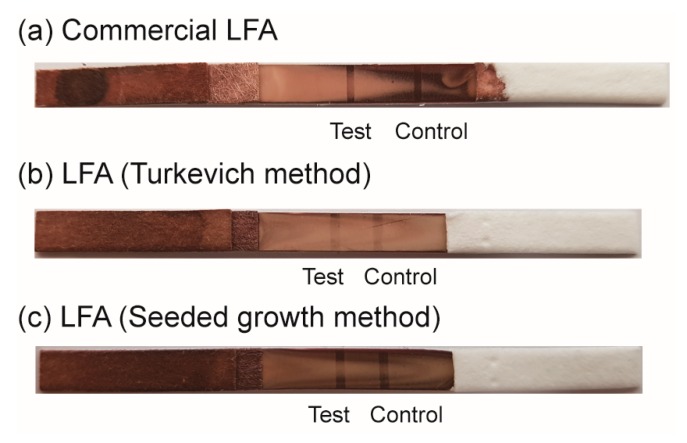
Photograph images of the practical test of LFAs ((**a**) commercialized HBV diagnosis kit (Humasis); (**b**) LFA based on Turkevich-Frens method; and (**c**) our LFA developed in this work) using human whole blood samples.

For practical tests, human whole blood samples (500 ng/mL HBsAg) were tested using three different LFAs, such as a commercialized HBV kit (Humasis), an LFA based on AuNPs obtained from the Turkevich-Frens method, and our LFA (42.7 ± 0.8 nm AuNPs). Two band signals were observed at three LFAs (Figure 7). Compared to the LFA based on the Turkevich-Frens method, our LFA shows more clear signals. These signals were comparable to the commercial LFA.

## 4. Conclusions

In this study, different sizes of AuNP-based LFA biosensors were developed for the detection of hepatitis B surface antigens. A seeded growth method was employed to prepare AuNPs with a well-controlled size of 34.0 ± 1.2 nm to 137.8 ± 0.45 nm. Different-sized AuNPs were characterized based on TEM images and UV-vis spectroscopy. To prevent AuNP aggregation with Ab, conjugation conditions were optimized at pH = 9 and 10 μg/mL. LFA biosensors were fabricated using five different-sized AuNPs and the color intensity was analyzed. The 42.7 ± 0.8 nm AuNP-based LFA biosensor showed the greatest sensitivity for detecting HBsAgs.
